# Age-Friendly Cities in Latin America: A Human Ecological Framework

**DOI:** 10.3390/geriatrics8030046

**Published:** 2023-04-25

**Authors:** Jonathan R. Guillemot, Mildred E. Warner

**Affiliations:** 1 Escuela de Medicina, Colegio de Ciencias de la Salud, Universidad San Francisco de Quito USFQ, Campus Cumbayá and Hospital de Los Valles, Quito 170901, Ecuador; 2 Instituto de Medicina Social & Desafíos Globales, Escuela de Salud Pública, Colegio de Ciencias de la Salud, Universidad San Francisco de Quito USFQ, Campus Cumbayá, Casilla Postal 17-1200-8414, Quito 170901, Ecuador; 3 Department of City and Regional Planning, Cornell University, Ithaca, NY 14853, USA; mwarner@cornell.edu; 4 Department of Global Development, Cornell University, Ithaca, NY 14853, USA

**Keywords:** age-friendly community, Latin America, WHO

## Abstract

Despite the demographic aging of Latin America, the uptake of the WHO’s Age-Friendly Cities Framework remains extremely low, with the notable exceptions of Chile, Mexico and Brazil. We argue for a broader human ecological framework, which focuses on the macro, meso and micro levels, to better address the context, challenges and opportunities for age-friendly cities in the Latin American region. The WHO’s age-friendly city domains are primarily at the meso (community) scale, with a focus on built environment, services and participation. We call for more attention to be paid to the macro policy scale to address concerns regarding migration, demography and social policy context. More attention also should be given to the micro scale to recognize the critical role of family and informal care supports. It is possible that the WHO domains are the result of a design bias, with Global North settings in mind for their development. We find the domains of UNICEF’s Child-Friendly Cities Initiative, which give more attention to the realities of the Global South, helpful to broaden the WHO’s Age-Friendly Cities Framework.

## 1. Introduction

In 2007, ahead of the launch of the Decade of Healthy Aging, the World Health Organization (WHO) established the Global Network of Age-Friendly Communities and Cities. The WHO framework encourages local governments to establish policies and actions favoring inclusiveness and social participation of older adults in society using participatory and civic involvement [1,2,3,4]. The Global South, and particularly Latin America, have displayed low uptake of the WHO age-friendly community framework. To this day, 1446 cities and communities have worked toward completing the requirements and entered the network, but the global distribution is uneven [5]. While Europe and North America are hubs of age-friendly cities, Latin American and African regions essentially remain deserts. According to the map of the WHO Global Network for Age-Friendly Cities and Communities [5], only a quarter of the network’s cities and communities belong to the Global South. Latin America accounts for 360 cities distributed as follows: 62 cities in northern Latin America (Mexico), 23 in Central America, 3 in the Caribbean and 272 in South America (see Table 1). While 23 countries report no members, Chile alone comprises 215 members, representing 60% of all Latin American members of the network. The northern Andean region (Venezuela to Bolivia and including Colombia, Ecuador and Peru) is particularly void of members, with a total of five local governments included. While Uruguay reports a single member (the City of Montevideo), it includes more than half of the country’s population.

The literature offers few explanations for the network’s low uptake in Latin America. One reason might be the lack of resources to implement the Vancouver protocol [6]. Barriers to access include a lack of information and community resources. The low number of WHO age-friendly members in the Global South could be due to a lack of awareness of the network’s existence, in part due to the lack of affiliates promoting the network, or the lack of perceived relevance [7]. Regions with low uptake do not reject the implementation of age-friendly policies, but low uptake suggests there may be barriers to entering the global network. A recent systematic literature review of processes, facilitators and barriers to accessing the network did not mention Latin America, suggesting a lack of research in the region [8]. The lack of age-friendly initiatives in Latin America limits research on the topic. The WHO [9] has given special attention to core indicators to promote equity and address both the physical and social environment to ultimately support better health and wellbeing in countries in both the Global North and the Global South. More evidence is needed to describe these barriers and develop proposals to promote an adaptation of the WHO framework to overcome these issues [10].

Fitzgerald and Caro identify barriers to the implementation of the eight WHO domains in cities which are already part of the network, but they do not discuss the barriers to cities attempting to access the network [11,12]. Barriers of implementation include a lack of economic resources for health and social services, as well as a lack of more general age-friendly city initiatives. Barriers to implementation have been studied in regions where local governments are already en route to age-friendliness, as illustrated by Neville and colleagues’ study in New Zealand [13] or by Rémillard-Boilard and colleagues’ worldwide study of 11 cases, which included 2 Global South cities that are located in Mexico and Chile, being the two Latin American countries with the most age-friendly cities [14]. A 2017 international survey of age-friendliness conducted by the AARP in the US and the International Division of the American Planning Association of 405 planners from the US and 154 from around the world found that non-US respondents were more likely to engage in facilitating practices and less likely to have local government support, but were more likely to report external motivations (national policies in support) and political barriers (ageist and gender bias) [15]. The study found engagement strategies were key, a finding which was also confirmed in prior research [16,17]. Black and Oh, in their evaluation of US age-friendly communities, emphasized the importance of local leadership and governance, but they also identified the need for more involvement of older adults and the monitoring of community progress [18]. 

To better understand the low uptake of the WHO’s Age-Friendly Cities Network in Latin America and to identify new actions to promote age-friendly action, this study analyzes the WHO domains from the perspective of the human ecological framework. 

## 2. Methods

We hypothesize that the low uptake of the WHO Age-Friendly Cities Framework in Latin America is in part caused by the lack of a holistic view of age-friendliness relevant to the Global South. We use the human ecological framework to identify potential limitations of the WHO Age-Friendly Cities Framework, which could be causes of the network’s low uptake in Latin America. We then propose the addition of elements to the WHO framework, which could lead to increased uptake in the future. 

The human ecological framework is a powerful theoretical framework used in social sciences to comprehend the complex relationship between humans and their environment, as it posits that human behavior and social systems are shaped by interdependent physical, social and cultural environments [19]. The human ecological framework typically includes three levels of analysis: the micro (individuals and families), meso (organizations and groups) and macro (policy and institutions) level. A wide range of health studies have used this framework to understand the relationship between the physical, social, economic and political environment of the population and how it affects the health and wellbeing of individuals and communities [20,21]. The human ecological framework posits that these levels interact and influence one another, and that the health and wellbeing of individuals is determined by the complex interplay between these different levels of organization. 

The human ecological framework has been used to demonstrate ways to address social determinants of health [22]. In the Latin American region, it has been used to better understand how macro trends and policies affect meso-level institutions and, in turn, micro-level family strategies [23,24]. We argue that a holistic approach, illustrated by the human ecological approach, may benefit age-friendly initiatives in the Latin American context.

We position the various domains of the WHO framework within the human ecological model’s threefold matrix of the micro, meso and macro levels. We find that most of the WHO domains are focused at the meso level. We then explore macro trends and policies and implications for micro-system (family) behaviors. Implications for the meso (community) layer, where the WHO domains are primarily focused, are then discussed. By looking at the WHO domains in the context of the human ecological framework, we can consider how to broaden the WHO framework to holistically address age-friendliness in Latin America.

## 3. Analysis

### 3.1. The Meso System and the Eight WHO Domains

When cities and communities commit to become age-friendly, the protocol of Vancouver promotes the implementation of eight WHO domains [6]: community and healthcare; transportation; housing; social participation; outdoor spaces and buildings; respect and social inclusion; civic participation and employment; and communication and information. As reported by the 2007 WHO report [1], the intention was to focus on the community level, which translated into the idea of a network of cities. This was supported by a significant body of evidence linking active aging with attributes associated with the city level [11]. Thus, the domains associated with the implementation of the WHO framework are primarily associated with the meso level. The original WHO Global Network of Age-Friendly Cities and Communities included 33 cities from 14 countries, but the only city members from the Global South were Zapopan, Mexico and Beirut, Lebanon [25]. While the focus groups held by the WHO in 2006–2007 included representatives from Central and South America and the Caribbean, the majority of participants were from the Global North [1].

The work linking these domains groups them into three arenas for action: the built environment (land use and outdoor spaces), services (housing, healthcare and transportation) and social engagement (participation, respect, civic participation and communication). In the US, for example, primary attention has been focused on the physical layer, as seen in the American Planning Association Guide for Age Friendly Communities [26]. However, research on rural communities has shown that the physical layer is not enough, and more attention needs to be given to services [27,28], social engagement [29,30,31] and the role of local government [20,32]. Social engagement may be especially important in rural communities, where the built environment and services are lacking [33]. Recent work has shown the critical importance of cross-agency collaboration in bringing these three layers together [18,32,34,35,36].

### 3.2. The Macro System in the Latin American Context

A rapidly aging demographic, high levels of out-migration and limited social welfare policies are relevant macro contextual factors for age-friendliness in Latin America and the Andean region. The Latin American region is aging at a fast pace [37] and it is no longer true to describe the region as young. When comparing age pyramids [38], Latin America is currently slightly older than the global average, while in 1970, the subcontinent was significantly younger. By 2050, Latin America is projected to be significantly older than the world average. Latin America is undergoing a speedy aging process [39]. Yet, the idea that Latin America is a young region persists, illustrated by the persistence of family planning programs targeting a reduction in fertility [40]. However, fertility has steadily declined from about 5 births per woman in 1970 to 1.9 in 2020, which is under the replacement rate, with there being few signs of reaching a plateau [38]. 

In addition to fertility trends, Latin America is markedly affected by national, regional and global migration patterns, which intensify the demographic phenomenon of aging. More specifically, we consider three sub-phenomena associated with human mobility: young adult outgoing migration and urbanization, limited overall incoming migration and specific older adult incoming migration [41]. At the national scale, Latin America is becoming more urban, a phenomenon contributing to the separation of families between urban and rural, as older adults are often left in rural areas [42]. Globally, Latin America is marked by out-migration, primarily toward North America and Europe. In comparison with increasing out-migration (from over 10 million in 1990 to over 30 million in 2020), immigration is stagnant and extremely low (under 3 million over the same period), making the balance sheet negative in nearly all countries [41]. Migration is somewhat compensated by regional migration that mainly occurs from Venezuela to Andean countries [41]. Migrants toward North America and Western Europe are essentially younger adults, with men seeking lower-qualification jobs and women often providing skilled and unskilled caregiving services to the aging population in the Global North [43]. Although insignificant in comparison to overall numbers, there is an increasing trend of immigration of older North American adults to Latin America seeking more affordable lifestyles, especially in countries such as Ecuador and Costa Rica [44]. This could create more pressure to address age-friendliness in the Latin American context.

Beyond demographic aging and the out-migration of younger adults, Latin America also suffers from underdeveloped social welfare systems. Access to social security is limited primarily to those in paid, formal employment, which comprises a small fraction of the population of older adults. Overall, in Latin America about 50% of adults over 65 years old have access to a pension, but there is significant diversity in the region (under 30% in Andean countries) [45]. In countries where there is a social security system, it is relatively new and suffers from economic pressures and privatization [46,47]. 

### 3.3. The Micro System and Its Importance in the Latin American Context

Given the lack of state-provided welfare services due to the economic limitations of many Latin American economies, much of the care provided to older adults comes from family solidarity. However, demographic aging, urbanization and out-migration make the family a less resilient system for providing care. Many Latin American countries suffer from what is understood as the global care chain or global care drain [48,49,50,51]. Young women migrate to Europe and North America, in part to care of older adults, leaving children to be cared for by grandparents at home. While remittance income is extremely important (at USD 126 billion, accounting for 2.5 percent of the Latin American GDP in 2021 (from a low of 0.11% in Chile to a high of 28% in Honduras) [52]), the social forms of care are strained. Family bonds are strong in Latin America, but they are stretched thin by the pressures of migration [48,49,50,51]. Thus, while family solidarity is more important in the Latin American context on the one hand, it is under especially heavy strain on the other, which raises challenges for age-friendly strategies in the region.

### 3.4. Macro and Micro Implications for the Meso System

These dual pressures from the macro and micro levels have important implications for the meso-system response. Dynamic outward migration patterns combined with aging demographic trends have multiple consequences: the acceleration of the aging process and the rapid shift of family and societal structures as well as solidarity systems. Compared to North America where demographic aging is slowed by constant immigration [53], the opposite is true in Latin America. In particular, the emigration of caregivers feeds into the care drain [54], which in turn, will leave local caregiving needs unmet. Migration affects the family structure and its associated solidarity network [48,49,50,51]. The adaptation capacity of families to migration is vastly different in these contexts. While not all solidarity is family-based, a significant proportion is, and the negative impact of migration is not easily replaced by other networks nor fully compensated by remittance income [55]. This is particularly true for older adults remaining in rural communities. This creates a vacuum of younger adults in certain areas, particularly in rural communities [56]. An accelerated aging process generates significant societal and cultural challenges. Instead of observing a demographic transition over a century or longer, the demographic transition will be complete over the course of about 50 years [57], which gives less time for society to adjust its education, industry, healthcare and policies [58]. This makes giving more attention to age-friendly approaches at the meso level all the more important.

In Global North settings, it may be expected that the community, or more generally the state, will take over when families fail to provide the necessary care for older members [59]. However, even in the Global North, high costs and challenges with access are key concerns [60]. Most Latin American countries do not have the financial strength to adequately support family solidarity and care [58]. In addition, prioritization of social action in parts of the Latin American region is challenged by the persistence of the lack of basic services such as electricity, drinking water, food security and infectious disease prevention [61,62,63]. The Latin American situation presents special challenges and barriers to the implementation of the WHO framework for age-friendly communities. 

At the macro level, it appears that the WHO framework assumes the presence of social welfare protections. This might explain why so little attention is given to macro-economic considerations in the WHO framework. At the micro level, the WHO domains do not specifically address the promotion of family solidarity (be it biological or of choice) for supporting aging in place, although this is mentioned as a subpoint under social inclusion [1]. This stands in contrast to the UNICEF [64] Framework for Child-Friendly Cities, which puts family at the core. While children are recognized as residing primarily in family networks, older adults are not, as illustrated by the comparison of domains between UNICEF’s child-friendly cities and WHO’s Age-friendly cities programs (Table 2). However, in Latin America, the family network remains a critical micro context, even if one under stress.

At the meso level, the provision for basic needs such as clean water is identified as key in the UNICEF framework. However, the WHO framework was largely silent on these until more recently [9]. We think this may be due to the initial formation of the WHO domains in the context of the Global North, where services for basic needs were assumed to be present. In contrast, the UNICEF Child-Friendly Cities Framework was explicitly developed with the Global South in mind [64]. The WHO Age-Friendly Cities Framework would benefit from having a broader set of domains. For example, basic services such as water, electricity and waste collection should be included, as should attention to food security and food sovereignty, as this is a critical need. This was considered as a missing dimension in the African context in the 2018 WHO report [65].

The 2015 update of the WHO framework [9] (p. 28) noted that, “The key principles which are reflected in the core indicators are equity, accessibility and inclusiveness.” Using these principles as a guide, this update can help countries in the Global South identify additional core domains that need attention. For example, the 2015 WHO report emphasized culture as an important resource. When we think how macro, meso and micro systems interact, this exposes new possibilities for action. Age-friendly communities should reflect the cultural backgrounds of individuals and their communities, and this may offer new possibilities for healthcare approaches. 

Three of the WHO domains refer directly or indirectly to the notion of socialization for older adults: social participation, respect and social inclusion, and civic participation and employment. The 2015 WHO update also paid special attention to economic security. However, the WHO domains are still relatively silent on the role of family, whether biological or social. Age-friendly policies could support the development of family alternatives for care, with particular attention being given to the unique context of Latin America. For example, in Ecuador, the ‘bono’ system established under the Correa regime in the 2010s paid family caregivers to support older adults and people with disabilities [24]. It was then picked up as a potential model for other middle-income countries in the region. 

Safety is another concern that was articulated clearly in the UNICEF Child-Friendly Cities Framework, and more recently, in the WHO Age-Friendly Cities Framework [9]. Safety is not just a concern for children, but is also a concern for older adults, where it has been found to be a key factor explaining physical activity [20]. Safety is an issue in Latin America in a way that is quite different from Global North realities. For example, fear of robbery or abuse, particularly in urban areas, has been exposed as a significant issue for the development of an age-friendly environment in the Argentinian context [66].

Most importantly, we need to give more attention to the capacity of local government. The meso level is deemed a primary target for the WHO Age-Friendly Communities Network. However, this assumes adequate macro-level and micro-level supports. In the Latin American case, this cannot be assumed. Direct service delivery by the local government sector is relatively recent in Latin America, as many services were national or regional in scope until the decentralization efforts of the 1990s [67,68]. How much capacity does the local government sector have to lead age-friendly approaches in the Latin American context? For example, with the Chilean situation, the network’s uptake is high as it aligns with more established social welfare policies, i.e., greater support from the state for an improved meso situation [69]. This regional discrepancy further supports our argument that increased attention to the macro system may enhance accessibility to the WHO network.

## 4. Discussion

In this paper we have explored possible reasons why the age-friendly initiative has not seen much uptake in Latin America. We argue that age-friendliness should be a fundamental component for planning in resource-limited contexts and not just a “luxury” to be addressed only after basic needs are met. This is especially important in Latin America where aging is an ongoing process which cannot be slowed or avoided. Thus, an age-friendly approach would help address the demographic transition and build toward a healthy aging society. Secondly, addressing the limitations of food security and food sovereignty, access to clean water and electricity, and disease prevention will take time, and may take possibly longer than the demographic transition will. 

Age-friendliness is influenced by multiple levels of social organization, including the individual, family, community and society levels. We argue that a holistic approach, illustrated by the human ecological framework, would benefit age-friendly approaches in the Latin American context. Migration patterns and the associated care drain, combined with the fast-paced aging of the region, are fundamental issues, which make age-friendly work all the more important. The limited-resource context, especially regarding macro-level social security supports and meso-level government capacity, restricts the implementation of the WHO framework in Latin America. However, heavy reliance on micro-level solidarity networks, such as families (biological or of choice), is also constrained by forces of migration and limited state support. More attention should be given to ways to promote such solidarity networks, especially through the participation of older adults to strengthen such networks. Even in the Global North, efforts are being made to support family networks as a means to achieve a more sustainable demographic transition [70,71]. 

**Figure 1:**
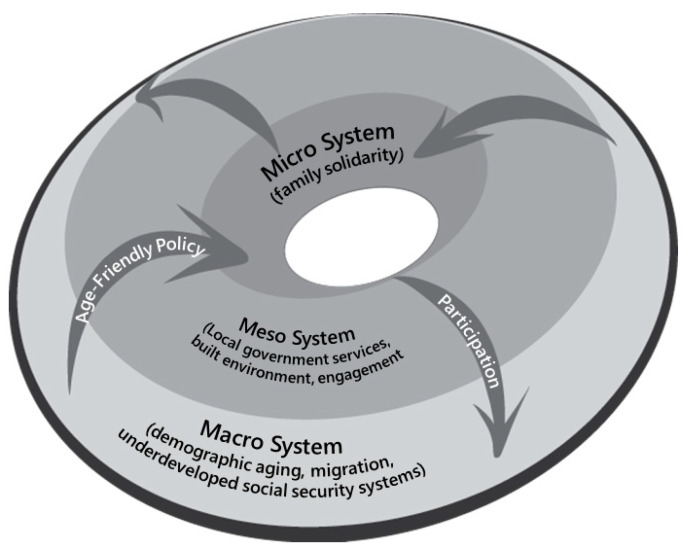
Representation of the human ecological framework in the context of age-friendliness. Author analysis; figure adapted from [24] and created by authors (MW).

The WHO Age-Friendly Cities Framework needs to expand its focus beyond the community to the national level to address macro constraints such as migration, demographics and social welfare policy. It also needs to give more attention to the micro level, particularly the role of family caregiving. The interaction across scales—macro, meso and micro—illustrates limits as well as new possibilities for action [23,24]. It is this dynamic network of flows that may yield new opportunities. This framework also may help shift aging from a deficit frame (cost to society and a passive group in need of services) to an activist frame based on the promotion of inclusivity, empowerment and engagement (Figure 1).

Bringing a human ecological approach to the framework of age-friendly communities will help policymakers pay attention to macro realities and constraints and the implications of a meso-level community response. The meso level can be a unifying construct to explore pathways that link to the micro and macro scales, providing avenues for change over time [72]. However, community-level capacity constraints are real, especially in contexts where macro-level state support is limited. Additionally, micro-level family support is strained. However, there are strategies that can be employed. These require an expansion of the eight WHO domains to include the macro context (demographics, migration and social welfare policy) and understanding the implications for basic services, as well as more attention being paid to safety and the role of family networks. By linking the UNICEF domains with the WHO domains, we believe a framework more appropriate for communities in Latin America may be developed. In lieu of seeing age-friendliness as an unreachable goal due to limited resources, a broader multi-level approach to planning can address the unique challenges communities face in Latin America.

At the meso level, local governments could strengthen the role of family caregiving with the following strategies: support family caregiver training and certification, and promote the constitution of local family solidarity networks so that caregiver respite becomes possible. Local governments cannot be the only source of palliative care for inadequate national policy frameworks. Age-friendly cities, in collaboration with other levels of government, could contribute to the recognition of the role of family caregivers. For example, even though family caregivers are unpaid, their work could be recognized and supported by national pension schemes (as occurs in some European countries). Possibilities exist, and thus, we just need to expand our view to a broader human ecological framework that recognizes the potential contributions of the national, regional and local levels of government to create a more age-friendly society.

## 5. Conclusions

This paper began with the question of why there has been such low uptake of the WHO Age-Friendly Cities Framework in cities in the Latin American region. We used a human ecological framework to show the need to take a multi-level approach that recognizes the importance of macro-level trends and constraints, as well as micro-level approaches. These have implications for the meso-level community strategies that lie at the heart of the WHO domains. By taking a holistic human ecological approach, we demonstrate the limitations of the WHO framework but also outline some future directions for action. This broader human ecological framework may have relevance beyond Latin America for communities across the Global South, helping to identify strategies to promote age-friendliness.

## Figures and Tables

**Table 1:** Latin American region: Cities in the WHO Age-Friendly Cities Network.

Latin American Regions	Countries	Number of Members (*n* =)
Northern Latin America (Mexico)	Mexico	62
Central America	Costa Rica	23
	Belize, El Salvador, Guatemala, Honduras, Nicaragua, Panamá	0
The Caribbean	Cuba	3
	Antigua and Barbuda, The Bahamas, Barbados, Dominica, Dominican Republic, Grenada, Haiti, Jamaica, St. Kitts and Nevis, St. Lucia, St. Vincent and the Grenadines, Trinidad and Tobago	0
South America	Argentina	18
	Bolivia	1
	Brazil	32
	Chile	215
	Colombia	3
	Paraguay	1
	Peru	1
	Uruguay	1
	Ecuador, Guyana, Surinam, Venezuela	0
Total		360

Source: Author analysis of the World Health Organization Global Network for Age-friendly Cities and Communities. https://apps.who.int/agefriendlycitiesmap/ (accessed on 17 March 2023).

**Table 2:** Comparison of domains between UNICEF child-friendly cities and WHO age-friendly cities. Author analysis.

UNICEF Child-Friendly Cities	WHO Age-Friendly Cities
Basic Services	Housing, Transportation
Safe Water	Community Support and Services (Health)
Safe Streets	Outdoor Spaces and Buildings
Opportunity to Play	Communication and Information
Civic Participation	Civic Participation and Employment
Family Support	Respect and Social Inclusion
Protection from Exploitation	Social Participation

## Data Availability

Not applicable.
